# How can we weather a virus storm? Health prediction inspired by meteorology could be the answer

**DOI:** 10.1186/s12967-021-02771-z

**Published:** 2021-03-09

**Authors:** Roberto Buizza, Enrico Capobianco, Pier Francesco Moretti, Paolo Vineis

**Affiliations:** 1 Istituto Scienze Della Vita, Scuola Superiore Sant’Anna, Piazza Martiri della Libertà 33, 56127 Pisa, Italy; 2 University of Miami, Institute of Data Science and Computing, Miami, FL US; 3 Italian National Council of Research (CNR), Liaison Office in Brussels, Brussels, Belgium; 4 Imperial College, London, U.K.

**Keywords:** Weather models, Health models, COVID-19, Monitoring and prediction, Complex systems, Predictability, Uncertainty

## The need for world health predictions

Operational weather predictions have shown us how a process-based approach relying on four key building blocks helps weathering storms. Nowadays, weather forecasts allow to predict events and enable planning relief actions few weeks in advance Hoskins [5], Buizza and Leutbecher [2]; Siilmann et al. [9].

We propose the establishment of a Health Prediction Center (localization site to be decided), with the mission to generate knowledge-based, reliable, skilful probabilistic predictions to help managing health-related risks. The Center should be global in scope, given that health challenges can impact at global scales (see,.e.g., the COVID-19 pandemic).

As for weather, health predictions should be probabilistic rather than deterministic ones (a ‘yes’ or ‘no’ forecast scenario), to recognize the fact that they are affected by uncertainties (e.g. due to initial and observation errors, poorly simulated multicausality, and model approximations) that have to be estimated. The Center will rely on a state of the art model and a systemic approach, and will address the increasing call for a more systematic use of mathematical models, *‘.. to assess available intervention options effectively and therefore indirectly for those deciding on and implementing public health policies..’*, [6].

Whoever tried to use the available data to monitor the spread of the COVID-19 epidemic in different parts of the world, realized that often the same variable (e.g. the number of infected cases) was measured differently in each country. This made the comparison of the spread in different countries uncertain, and also made extremely difficult to understand whether control and prevention measures taken in one country could have an effect in another. Robust predictions were consequently very rare.

This scenario retraces the one for weather predictions in the 1960–70s: few observations were collected, often with different methods, very few were exchanged, only simple weather models were developed [4, 10]. People were still rather sceptical about any reliability of weather forecasts for more than few days. A big step forward occurred in the 1970s, when the World Meteorological Organization (WMO) promoted the adoption of standards in data collection and exchange, and it established the Global Telecommunication System (GTS) to co-ordinate data exchange. Meteorological services started developing their own models and operational forecasts. The European Union established the European Centre for Medium-Range Weather Forecasts (ECMWF), to address weather prediction with the support of numerical methods. Nowadays, numerical weather forecasts are a fundamental source of extremely valuable information for a wide range of human activities [1–3], providing decision makers with standardized, high-quality data for scenario analyses able to assess the impact of interventions.

## The four key processes of a prediction system

Analogously for weather, any prediction system should include four key processes:

1. *Observe,* to identify relevant variables to estimate the state of the system, to define measurement standards, to collect continuously and exchange them in real time.
2. *Interpret and model*, to understand which are the main drivers, how different system components interact, and how phenomena with local or global scale can affect its dynamics.
3. *Estimate and predict*, to have an accurate knowledge of the current state of the system, by optimizing the information extracted from the observations with a first-guess of the state of the system defined by a short range forecast, and to forecast how it will evolve.
4. *Diagnose and verify*, to understand whether the model is capable to reproduce realistically the system dynamics, and to inform the users of the quality of the predictions, measured using agreed metrics and standards.

The continuous loop between these four main processes helps refining the prediction system, and generate increasingly valuable forecasts.

Considering the modelling aspects, we do not expect that health systems follow laws similar to the ones that describe the behaviour of the Earth system components that determines its weather and climate. In health systems, the level of complexity is higher because humans can modify the determinants of diseases and protect themselves, thus interfering with the spontaneous dynamics of the system in a way that does not occur for weather. Health models need to take also these aspects into account.

## The key players of a prediction infrastructure

In weather, the meteorological infrastructures rely on organizations that work at three different levels: the World Meteorological Organization (WMO), international centers such as ECMWF, and national meteorological services. The WMO aims to coordinate development and promote coordination and collaboration at the global level. International centers such as ECMWF in Europe, and few of the national meteorological centers issue global forecasts, while the vast majority of the national meteorological centers focus on the local scale and the short-range. These forecasts are generated by nesting the limited-area high-resolution models in the global forecasts, which provide them with the initial and boundary conditions necessary to drive the limited area models.

**Fig. 1:**
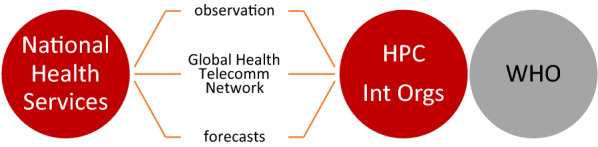
The World health infrastructure: schematic of the interactions between the National Health Services (NHSs), the proposed Health Prediction Center (HCP) and other international organizations, and the World Health Organization. WHO, with the support of the NHSs and the HPC, defines the type of observations to collect, their standards, and a communication protocol. The NHSs collect daily observations, and exchange them in real time via the health global telecommunication network (GHTN). The HPC has real-time access to these data, produces global analyses (i.e. snapshots of the actual health state of the globe) and forecasts, and issue them. Forecasts and analyses are also exchanged via the GHTN. The NHSs have access to the HPC global analyses and forecasts, and use them in their daily operational activities

A similar model based on three interacting layers is envisaged for health predictions with National Health Services and International Health Institutions, including the proposed Health Prediction Center, working in a coordinated manner to improve the flow of standardized data and the exchange of health predictions (Fig. 1).

## Parallelism between weather and health prediction

Table 1 aims to show the parallelism between the methodologies and actions followed in operational weather prediction according to the four key processes, and the ones that could be followed in health prediction. Similarities include the need for timely observations, although the observed variables, data platforms and methods are different. Data assimilation methods are also similarly, aimed at estimating the system's states at regular time intervals and to be used to initialize predictions for the future states. Ensemble-methods could be used in both scenarios to provide uncertainty estimates.

**Table 1:** Methodologies applied to complete the four key processes of numerical weather prediction, and proposed equivalents in health prediction

	Weather and climate	Health
Observe	Observed variables include wind, temperature, humidity, pressure, radiation, cloud cover,.. taken by different observation platforms (from land stations to satellites)	Observed variables include agents of disease (pathogens); health state of individuals and communities (hosts); environmental characteristics and triggers (host–pathogen interactions); collective phenomena inducing anomalous fluctuations
Interpret and model	The scientific relies on the laws of physics applied to a rotating fluid. The modelling is based on the numerical integrations of finite-approximations of the fluid equation. The models include parameterisation schemes that simulate most relevant processes, including stochastic terms designed to simulate, to the best of our k knowledge, processes that are poorly known, or that would be computationally too expensive to be treated explicitly	Health modelling (HM) is often multidimensional/multifactorial and targets typically the disease agent, the individual background conditions and the interactions mediated by genetic, socio-economic, lifestyle, legislative and environmental factors. HM is a complex multifaceted process that depends on interpreting and understanding contexts from which to acquire knowledge by gathering, assimilating and elaborating data and information to match complexity. Scientific understanding depends on the cross-linking between mathematical model formalism with biological and behavioural structures and dynamics
Estimate and predict	Estimates of the actual state of the system are generated by solving optimisation problems that use as input all available observations and model forecasts. Predictions of the future states are generated by integrating numerically the system equations on super computers. Ensemble methods are used to provide confidence intervals and uncertainty estimations	Estimates are subject to spatio-temporal dynamics evolving rapidly and thus requiring incremental modelling schemes that a) efficiently update the values driving health trajectories based on newly captured information and b) encompass evidence within context-driven scores and predictions to limit the general uncertainty
Diagnose and verify	Diagnostic studies are based on key events and on a sound statistical analyses of the model performance over many cases (say at least few hundred cases). Verifications of forecast quality rely on a range of metrics that provide relevant information to the forecast users and the model developers	The more data are available, the better it is. Diagnostics presents constrains (availability of tests, limited capacity etc.) that might affect model performance or at least explain the role of spatio-temporal factors in determining accuracy and reproducibility. Verification depends on the choice of metrics that must consider many data latencies and gaps and should be selected to fit multiple specific objectives, purposes and trade-offs (ethical principles, improving accessibility, reducing costs, connectivity, spatial aggregation, similarity and dissimilarity system-wide and at localized contexts etc.)

The uncertainty is associated with lack of knowledge about context (spatio-temporal diversity), data nature and models. In weather prediction, the starting points are the conservation laws of physics. In health modelling, a multifaceted process includes genetic, socio-economic, lifestyle and environmental factors. Before using models as predictive tools, identifying and quantifying the sources of uncertainty through well-chosen parameters are fundamental to limit the amount of bias to a sustainable level. In terms of performance measurement, parallelisms exists: for example, a metric developed to assess the quality of clinical trials, the Relative Operating Characteristic curve, has been adopted in weather prediction to assess the capability of a probabilistic forecast system to discriminate between the occurrence and non-occurrence of weather events.

## The key objectives of a Health Prediction Center

The Center should have the following key objectives:

i. To collect and give access to global health data (observations and predictions generated by different, national and international institutions, including its own);
ii. To monitor the world health state;
iii. To produce global forecasts;
iv. To carry out the scientific and technical research needed to improve health prediction models;
v. To coordinate and promote data and knowledge sharing;
vi. To provide access to objective diagnostic and verification of all existing health prediction systems;
vii. To support training and knowledge sharing for the benefit of science and decision-making.

The Center should produce and make available reliable and accurate global health analyses (which provide an objective, observation-based snapshot of the daily situation) and forecasts valid for weeks and months ahead. It should develop data archive capabilities, data analysis tools and methodologies, and provide training in health monitoring and prediction. Most importantly, the Center should build a reliable, realistic, state of the art, global, probabilistic prediction model. Let us stress that this probabilistic model needs to simulate all relevant sources of forecast error. As Saltelli et al. [8] pointed out, *‘Asking models for certainty or consensus is more a sign of the difficulties in making controversial decisions than it is a solution, and can invite ritualistic use of quantification. Models’ assumptions and limitations must be appraised openly and honestly.’*

The Center will liaise also with other international health agencies and institutions, and act as a one-stop trustworthy coordination point, where scientists and decision makers could find the most updated and accurate information. It will provide them with timely ‘global health analyses’, generated by merging all shared global data and short-term first-guess forecasts, and ‘global health forecasts’.

As it was the case for ECMWF in 1975, the European Union could now integrate the goals of digitalization and health services with the establishment of the HPC as a new, independent organization.

## Weathering the next health storm is possible

The Covid-19 pandemic has bound policy makers to an unprecedented challenge. Control and forecast are the two main aspects that drive the policy decisions in their planning and implementation phases. Here we looked at the prediction problem. We propose to reflect and learn from the past and from the experience of other fields, in particular from those experiences which have already encountered analogous challenges in tackling complex systems.

The case of weather has provided valuable insights, and a possible model to follow to build a structured, accessible, transparent and knowledge-based information and forecast system, which can be used to support health decisions and interventions. We are fully aware that the weather and health systems are different, but they are equally complex, and both require/benefit from predictions. Relevant accessible data and adequate models for providing robust predictions are a necessary step in supporting political negotiations and the adoption of specific interventions.

Weather affects almost all human activities, and today we can manage weather-related risks thanks to the availability of high-quality data and accurate and reliable predictions.

Health affects all human activities, as we have witnessed in the past few months. We believe that if we had the equivalent of the weather infrastructure, supported by realistic and reliable probabilistic prediction systems, better and more informed decisions could have been taken. Indeed, Poletti et al. [7] stressed that predictive modelling can be extremely valuable, provided that it is knowledge-based, as we discussed above, and called for a more systematic integration of predictive modelling into decision-making processes. We therefore call for an action aiming to establish a Health Prediction Center. This will develop a global health accessible prediction model, capable to contribute with accurate analyses and forecasts to the future health of the global population.

## Data Availability

Not applicable.
